# Supplementary material for: Evaluation of Lipopolysaccharide and Interleukin-6 as Useful Screening Tool for Chronic Endometritis
Source: Int J Mol Sci. 2024 Feb 7;25(4):2017. doi: 10.3390/ijms25042017 (PMC10888589; doi:10.3390/ijms25042017)
Supplement: Supplementary file 1 [file ijms-25-02017-s001.zip › ijms-2826834-supplementary.pdf]

	Control patients	Patients with suspected CE	*P-value*
**Number**	15	13	
**Characteristics**			
Age	35.1 ± 0.94(29-41)	35.3 ± 1.15(27-42)	0.91
BMI	25.6 ± 1.71	21.5 ± 0.67	0.17
Smoking, n (%)	2/15(13.3)	0/13(0.0)	0.48
**Plasma concentration**			
Estradiol (pg/ml)	279.1 ± 59.2	393.3 ± 85.3	0.24
Progesterone (ng/ml)	1.04 ± 0.20	1.09 ± 0.59	0.15
LH (mIU/ml)	23.8 ± 6.40	20.1 ± 6.68	0.83
FSH (mIU/ml)	11.1 ± 1.71	9.0 ± 1.35	0.37
**Supernatant concentration**			
Menstrual cycle	12.9 ± 0.63	21.6 ± 1.31	<0.05
Estradiol (pg/ml)	104.1 ± 18.09	41.7 ± 7.82	<0.05
Progesterone (ng/ml)	1.27 ± 0.27	6.29 ± 1.60	<0.05
**Vaginal culture**			
Lactobacillus spp., n (%)	4/15(26.7)	3/13(23.1)	1.00
Escherichia coli, n (%)	2/15(13.3)	2/13(15.4)	1.00
Others, n (%)	10/15(66.7)	7/13(53.8)	0.70

Table S1. Clinical characteristics of the patients. One patient with CE was not examined for plasma con-centration and vaginal culture. Abbreviations: BMI (Body Mass Index). The values are shown as mean ± SEM and P < 0.05.

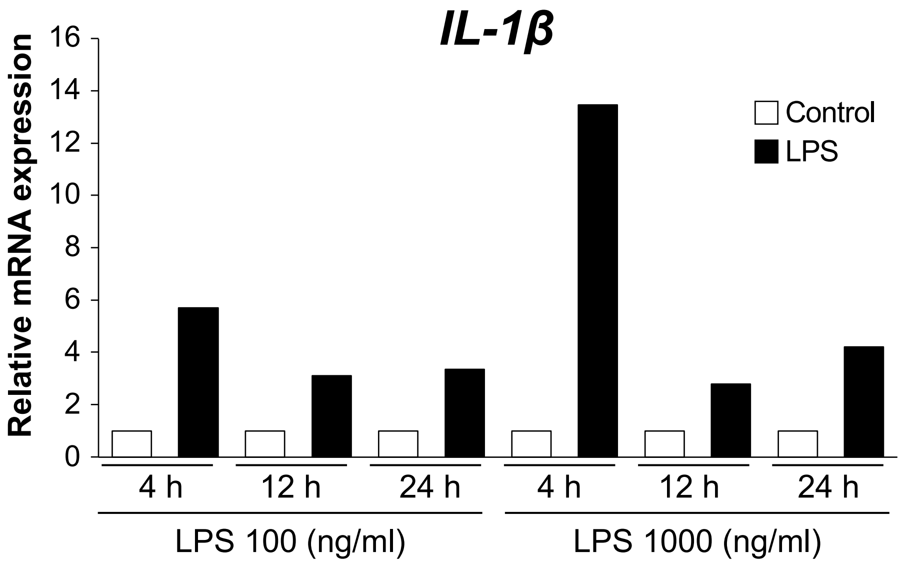

Figure S1. IL-1β gene expression for 4, 12, and 24 h using EM-E6/E7/hTERT-2 cells.
The cells were then treated with LPS (0, 100, and 1000 ng/ml). The experiments were conducted once.
The values are shown as the mean ± SEM.

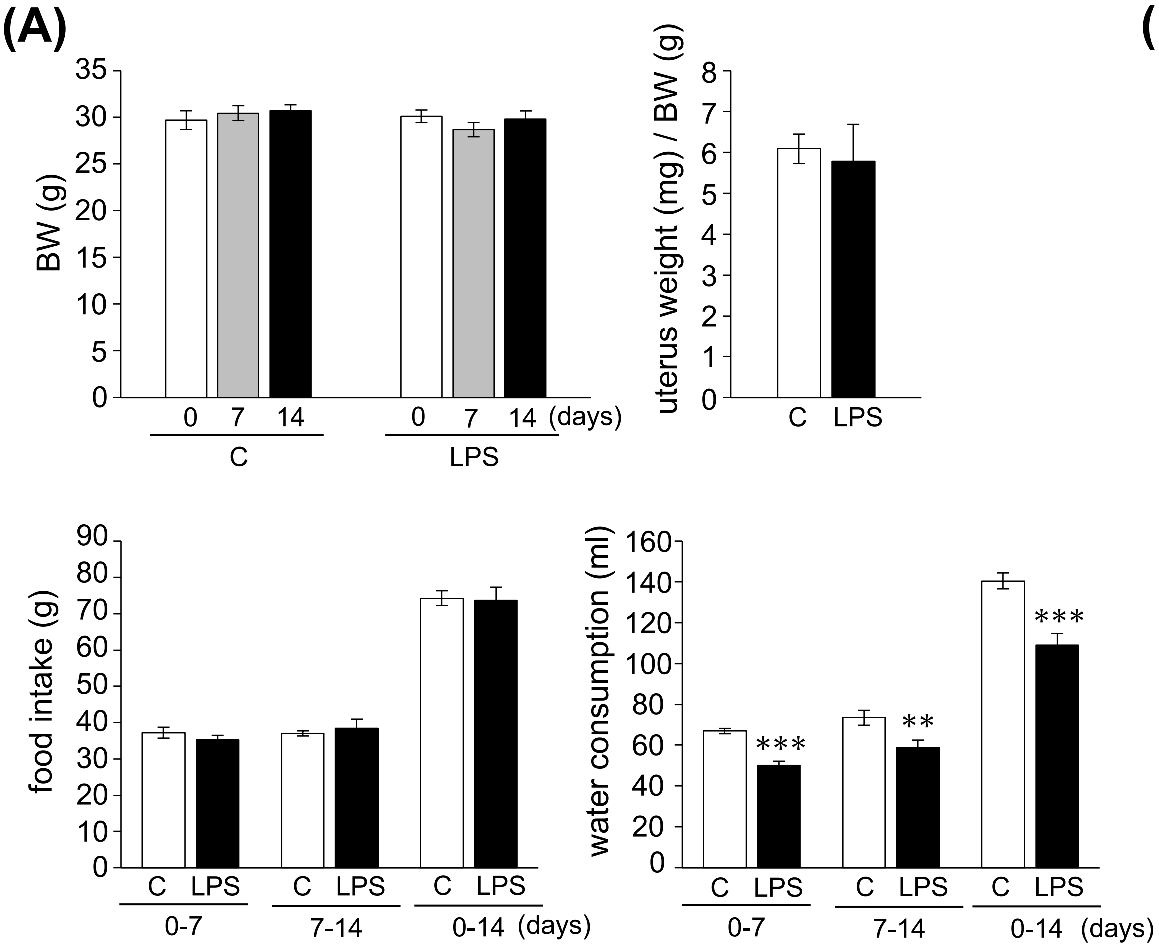

(B) **LPS concentration (ng/ml) in mouse endometrial tissue**

	Plasma	Uterine tissue
PBS 1	0.048	0.025
PBS 2	0.002	0.093
PBS 3	0.003	0.061
PBS 4	0.013	0.092
PBS 5	0.002	0.030
LPS 1	0.006	0.067
LPS 2	0.002	0.110
LPS 3	0.002	0.034
LPS 4	0.002	7.601
LPS 5	0.002	0.046

Figure S2. Physiological effects of LPS in mice (A) Mouse body weight (BW), uterine weight/BW, food intake, and water consumption (control, n = 5; LPS group, n = 5). (B) Concentration of LPS in plasma and uterine tissue. Values are shown as the mean ± SEM; ** $P < 0.01$, *** $P < 0.005$.

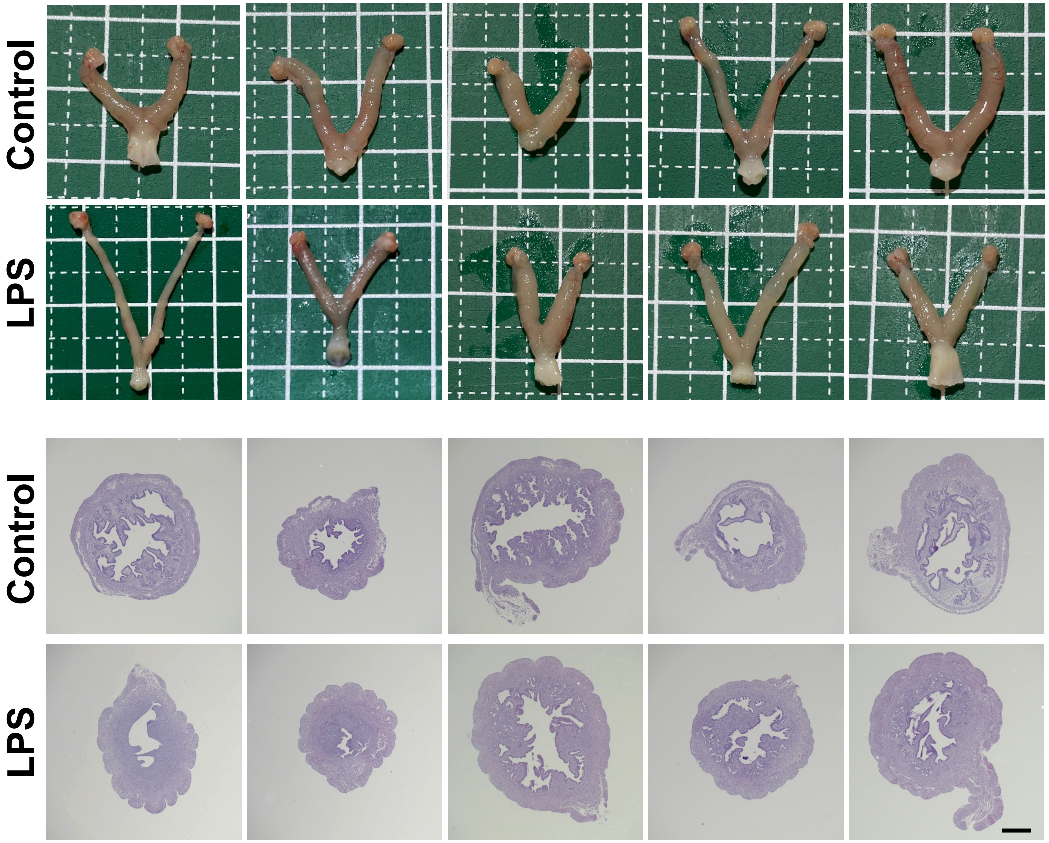

Figure S3. Histology of the uterus after PBS and LPS administration (control n = 5, LPS group n = 5). (×40) with Scale bar = 50 μm.

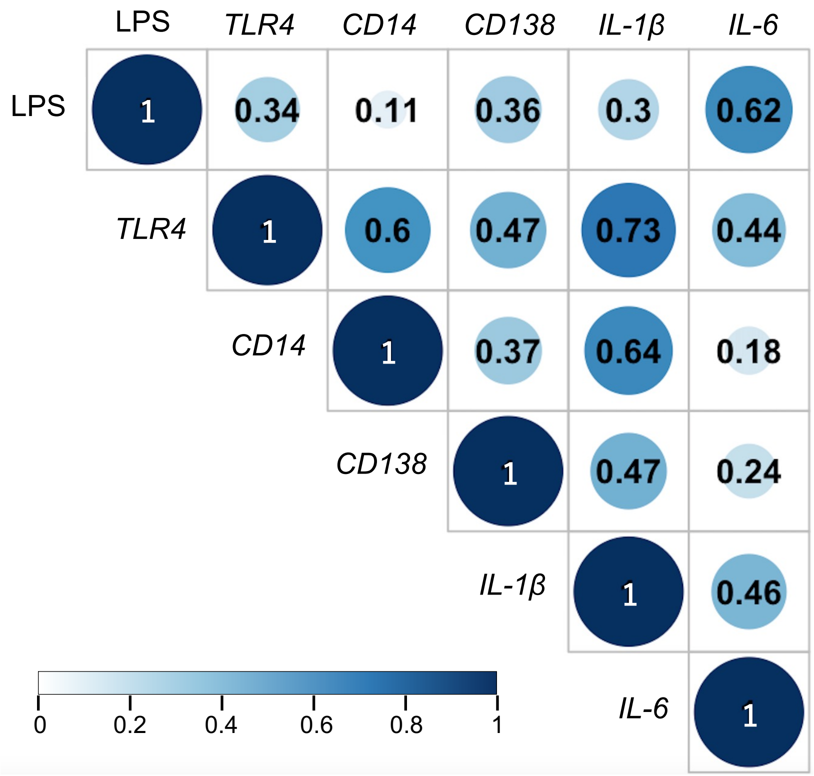

Figure S4. Correlation coefficients between LPS and inflammatory markers in human endometrial tissue (n = 23).

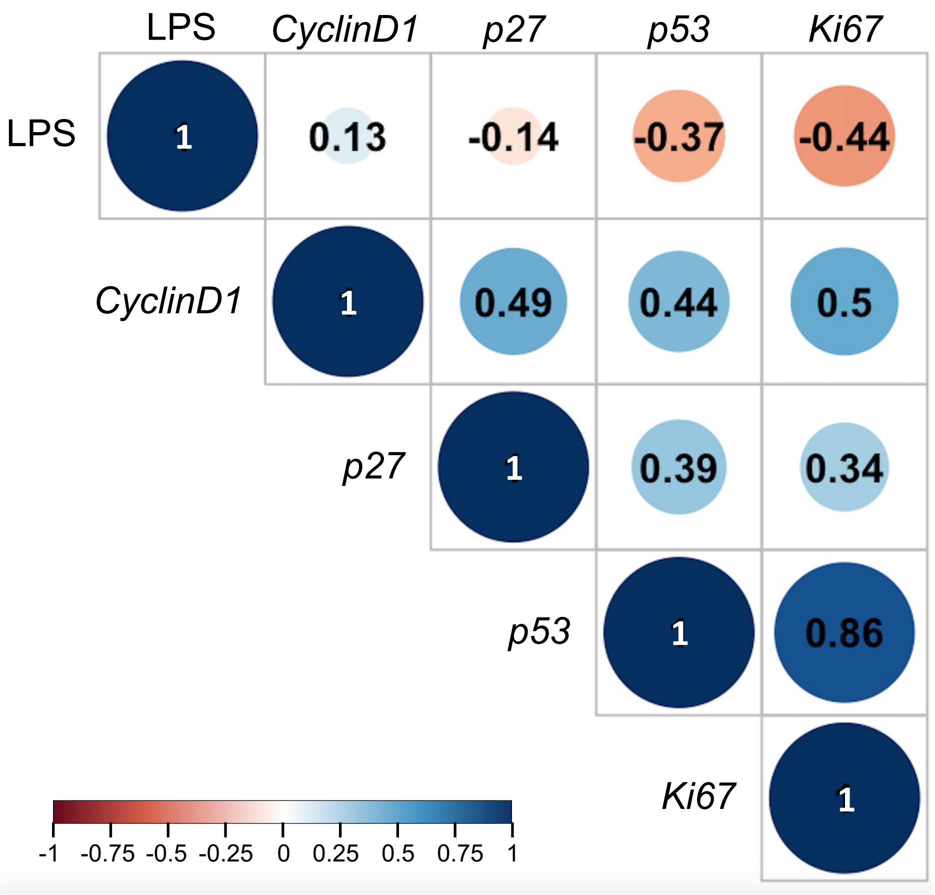

Figure S5. Correlation coefficients between LPS and cell cycle markers in human endometrial tissue (n = 23).
